# Dr. Cunningham’s Defense of the Hog

**Published:** 1888-12

**Authors:** 


### DR. CUNNINGHAM'S DEFENSE OF THE HOG.

We will not attempt a reply to Dr. Cunningham's argument; it were useless to do so, holding, as we do, such widely different views of what constitutes "filth." When the Doctor says "the hog will keep everything neat and tidy" about him, we can not help believing that he intends us to understand that he is speaking in a "Pickwickian sense," relatively; and, in this case, relatively to the—horse!—an animal, which, if we are not mistaken, is generally considered a model of cleanliness, and as "fastidious as a cat!" It seems a parody on the accepted views of sanitation and hygiene to confess that one's measure of neatness and tidiness is filled by the habits of the hog. If the *hog* is not *filthy*, what is? We thought, the world over that he was accepted as the very type of filth, and it has become a by-word and reproach—"as filthy as a hog." Queer ideas of neatness the Doctor holds, surely. But, leaving "habits" out of consideration,—the animal is the abode of loathsome parasites (in health) both within and without, which alone renders him unfit for human food; as unwholesome as the very offal which God doubtless created him to destroy. No, Doctor, the hog has not "come to stay." Another generation or two, with the progress of sanitary science, will see him disappear from the table of all who have correct ideas of hygiene, and consigned solely to scavenger work in company with his companions—the maggot and the buzzard.

---

It is very provoking, to find out, when too late to correct it, that Dr. E. J. Beall's name, at the head of his valued and valuable article is spelled "Bell" instead of "Beall." We make the correction, however, in the re-print, which will be sent out, and tender the Doctor an apology.

